# Immunomodulatory Effect of Novel Electrospun Nanofibers Loaded with Doxycycline as an Adjuvant Treatment in Periodontitis

**DOI:** 10.3390/pharmaceutics15020707

**Published:** 2023-02-20

**Authors:** Vlad Andrei, Sanda Andrei, Adrian Florin Gal, Vasile Rus, Luciana-Mădălina Gherman, Bianca Adina Boșca, Mihaela Niculae, Reka Barabas, Oana Cadar, Elena Dinte, Dana-Maria Muntean, Cosmin Petru Peștean, Horațiu Rotar, Antonia Boca, Andreea Chiș, Manuela Tăut, Sebastian Candrea, Aranka Ilea

**Affiliations:** 1 Department of Oral Rehabilitation, Faculty of Dentistry, “Iuliu Hațieganu” University of Medicine and Pharmacy, 400012 Cluj-Napoca, Romania; 2 Department of Biochemistry, Faculty of Veterinary Medicine, University of Agricultural Sciences and Veterinary Medicine, 400372 Cluj-Napoca, Romania; 3 Department of Cell Biology, Histology and Embryology, Faculty of Veterinary Medicine, University of Agricultural Sciences and Veterinary Medicine Cluj-Napoca, 400372 Cluj-Napoca, Romania; 4 Experimental Centre of University of Medicine and Pharmacy “Iuliu Hațieganu”, 400349 Cluj-Napoca, Romania; 5 Department of Morphological Sciences, Faculty of Medicine, “Iuliu Hațieganu” University of Medicine and Pharmacy, 400012 Cluj-Napoca, Romania; 6 Department of Infectious Diseases, Faculty of Veterinary Medicine, University of Agricultural Sciences and Veterinary Medicine Cluj-Napoca, 400372 Cluj-Napoca, Romania; 7 Department of Chemistry and Chemical Engineering of Hungarian Line of Study, Faculty of Chemistry and Chemical Engineering, Babeș-Bolyai University, 400028 Cluj-Napoca, Romania; 8 INCDO-INOE 2000, Research Institute for Analytical Instrumentation, 400293 Cluj-Napoca, Romania; 9 Department of Pharmaceutical Technology and Biopharmaceutics, Faculty of Pharmacy, “Iuliu Hațieganu” University of Medicine and Pharmacy, 400012 Cluj-Napoca, Romania; 10 Department of Surgery and Intensive Care, Faculty of Veterinary Medicine, University of Agricultural Sciences and Veterinary Medicine Cluj-Napoca, 400372 Cluj-Napoca, Romania; 11 Department of Cranio-Maxillofacial Surgery, Faculty of Dentistry, “Iuliu Haţieganu” University of Medicine and Pharmacy, 400001 Cluj-Napoca, Romania

**Keywords:** immunomodulator, doxycycline, periodontitis, electrospinning, polylactic acid, nano-hydroxyapatite, nanofibers, biomaterial, biomarkers

## Abstract

The immunomodulatory effect of a novel biomaterial obtained through electrospinning, based on polylactic acid (PLA) and nano-hydroxyapatite (nano-HAP), loaded with doxycycline (doxy) was evaluated in an animal model. The treatment capabilities as a local non-surgical treatment of periodontitis was investigated on the lower incisors of Wistar rats, after the induction of localized periodontitis using the ligature technique. Following the induction of the disease, the non-surgical treatment of scaling and root planing was applied, in conjunction with the application of the new material. The results of the treatment were evaluated clinically, using the tooth mobility and gingival index scores, as well as histologically. The salivary concentrations of matrix metalloproteinase 8 (MMP-8) and plasmatic concentrations of interleukin 1 (IL-1), interleukin 6 (IL-6) and tumor necrosis factor alpha (TNF-α) were also monitored. Two weeks after the ligature application, the periodontal disease was successfully induced in rats. The application of the novel biomaterial obtained through electrospinning was proven to be more effective in improving the clinical parameters, while decreasing the salivary MMP-8 and plasmatic IL-1 and TNF-α concentrations, compared to the simple scaling and root planing. Thus, the novel electrospun biomaterial could be a strong candidate as an adjuvant to the non-surgical periodontal therapy.

## 1. Introduction

Periodontitis is a chronic disease that affects the dental supporting apparatus, characterized by the progressive destruction of the gingiva, alveolar bone, and periodontal ligaments. Periodontitis has an inflammatory nature, with the initiation and progression linked to the subgingival biofilm [1].

In addition to the local effects of untreated periodontitis, which consist of an irreversible loss of alveolar bone and gingiva, a systemic impact of periodontitis has been proven [2]. A gross accumulation of bacteria in the oral cavity may lead to a general dissemination of pathogens; moreover, locally activated lymphocytes may cause a high systemic inflammatory status, thus aggravating pre-existing conditions [3]. Furthermore, research has demonstrated the bidirectional relationship between general health and periodontitis, with severe cases of periodontal disease that are rapidly evolving and refractory to treatment identified in patients with general pathologies [2,3].

Given the systemic effects and the importance of early diagnosis, the use of proteins and enzymes as biomarkers for the progression of the periodontal disease has been proposed. Matrix metalloproteinases (MMPs) naturally occur in the wound healing processes, and are also implicated in major inflammatory diseases, through collagenolysis and elastolysis [4]. Matrix metalloproteinase 8 (MMP-8) is released by neutrophils once they have been activated by proinflammatory mediators or damage-associated molecular patterns [5]. The periodontal ligament is rich in collagen fibers, of which 80% are type I collagen, and 20% are type III collagen, and both types of collagens are cleavable by MMP-8 [6,7]. MMP-8 concentrations in periodontal disease are higher than in healthy patients, suggesting that this proteinase is responsible for the cleavage of collagen fibres in the gingiva [8]. Thus, MMP-8 has been proposed as a biomarker for the early diagnosis in periodontitis [7,8,9].

Interleukin 1 (IL-1), interleukin 6 (IL-6) and tumour necrosis factor alpha (TNF-α) are proinflammatory cytokines linked to the initiation of periodontal inflammation and bone resorption [10,11]. Moreover, these cytokines limit the reparatory potential of periodontal tissues by inducing apoptosis in cells responsible for collagen synthesis [11]. Given their major roles in the early stages of periodontitis, and their variation in salivary and plasma concentrations, these molecules have been proposed and used as biomarkers for chairside patient screening [12,13].

Periodontitis may also have a negative effect on the patients’ quality of life. The irreversible loss of supporting tissues of the teeth may lead to aesthetic disfunctions, due to gingival retractions, as well as functional disorders, including difficult mastication due to teeth mobility and eventual loss. Moreover, while chronic periodontitis may be asymptomatic, gingival inflammation and bleeding cause pain and discomfort during normal activities, such as eating and oral hygiene [14].

The non-surgical periodontal treatment includes a complex series of procedures that aim to reduce the determining factor, microbial biofilm, and to eliminate the aggravating local and general factors, with the purpose of improving the periodontal status. The manual instrumentation of the periodontal pockets, by scaling and root planing, helps remove the subgingival calculus and biofilm, as well as the microbial endotoxin-penetrated cementum [15]. The limitations of the manual instrumentation reside in the inefficient debridement of deep periodontal pockets, due to the irregular radicular surfaces and the narrow furcation areas, which are inaccessible to periodontal instruments [16,17]. Given the infectious nature of periodontitis and the limitations of the non-surgical periodontal treatment, the use of local antibacterial and anti-inflammatory substances has been proposed [18]. However, due to the current lack of satisfactory long-term therapeutic outcomes, as well as the difficult application and deficient maintenance, the use of local antibiotics and antiseptics is limited [17,19].

The aim of the in vivo study was the evaluation of the immunomodulatory effect of a novel electrospun matrix system based on 5% polylactic acid (PLA) and nano-hydroxyapatite (nano-HAP), loaded with doxycycline (Doxy, 7 g/L) and topically applied in experimentally induced periodontitis in male Wistar rats. The novel biomaterial was used in combination with an inert mucoadhesive film (with a mechanical isolation role), as an adjuvant to mechanical instrumentation of the periodontal pockets.

The therapeutic outcomes were evaluated by monitoring the clinical parameters (teeth mobility and gingival index), assessing the salivary MMP-8 and plasma levels of IL-1, IL-6 and TNF-α, and histological examination of periodontium.

## 2. Materials and Methods

### 2.1. Development of the Electrospun Matrix System

The electrospinning method used for manufacturing the novel biomaterial, consisting of doxy loaded (7 g/L) electrospun nanofibers based on 5% polylactic acid (PLA), with a molar mass of Mw = 60,000, and nano-hydroxyapatite (nano-HAP), was previously published and the nanofibers were characterized by Fourier-transform infrared spectroscopy (FT-IR), thermogravimetry and differential thermal analysis (TG-DTA) and scanning electron microscopy (SEM). The in vitro release of doxy in simulated body fluid (SBF) and phosphate buffer solution (PBS) was also evaluated. In both media, doxy shows sustained release over 96 h. In SBF, an increasing dissolution tendency was observed with a decreasing release rate over time. The same phenomenon was also observed during the measurement in PBS, although a lower amount of dissolved active substance was detected [20]. The in vitro antimicrobial effect of the doxy-loaded nanofibers was previously demonstrated by the authors (Andrei V., et al., [21]), using a Kirby-Bauer disk diffusion susceptibility test. Moreover, the antimicrobial efficacy of the biomaterial against two periodontal pathogens, *Aggregatibacter actinomycetemcomitans* and *Porphyromonas gingivalis*, supports its potential use for the non-surgical periodontal therapy [21]. Thus, the in vivo evaluation of the material was required for the clinical validation of the novel biomaterial.

### 2.2. Mucoadhesive Film Preparation

The mucoadhesive film was formulated containing hydroxypropyl methylcellulose (Colorcon) and polyacrylic acid (B.F. Goodrich), usable in combination with doxy-loaded electrospun material, for topical applications in the periodontal pockets with a mechanical isolation role. Several formulations with different compositions were prepared using the solvent casting method and were in vitro characterized regarding the physicochemical properties. The formulation with the most favourable characteristics demonstrated in vitro, in terms of surface pH (6.3 ± 0.29), thickness (0.4 ± 0.001 mm), folding endurance (>350), as well as ex vivo bioadhesion retention time (280 ± 15 min) was selected for further in vivo applications, in combination with the novel electrospun biomaterial.

### 2.3. Study Design

The present study was conducted in the Laboratory Animal Facility—Centre for Experimental Medicine, “Iuliu Hațieganu” University of Medicine and Pharmacy, Cluj-Napoca. The experimental protocols had been previously approved by the Institutional Ethics Committee of the University of Medicine and Pharmacy “Iuliu Hațieganu” Cluj-Napoca (No 47/1.03.2021), as well as by the Romanian National Sanitary Veterinary and Food Safety Authority (No 266/28.06.2021). The present experimental study was conducted in accordance with the Romanian laws regarding the ethics and welfare of experimental animals (Directive 86/609 EEC/1986; Romanian Law 205/2004; Romanian Law 206/2004; Romanian Law 471/2002; Romanian Law 9/2008; Romanian Order 143/400).

The in vivo immunomodulatory effect of the novel biomaterial was evaluated by topic application for the treatment of experimentally induced periodontitis in Wistar rats. The subjects were purchased, stored, and treated in the Laboratory Animal Facility—Centre for Experimental Medicine, “Iuliu Hațieganu” University of Medicine and Pharmacy Cluj-Napoca. The animals were kept in a closed-circuit environment and were housed in plastic-bottomed cages with stainless-steel bars (Tecniplast Buguggiate, Italcages, Varese, Italy), fitted with adequate ventilation, at a temperature between 20–24 °C and a relative humidity of 55% ± 10%. The food, consisting of granulated combined fodder and biologically pure water, were provided *ad libitum*. The cages were lined with dry, absorbent, non-toxic and non-infectious animal bedding.

The experimental periodontitis was induced using the ligature technique. A 5/0 multi-thread silk suture (Silk Braided, Lux Sutures, Weiswampach, Luxembourg) was placed around the lower incisors of the specimens, with the purpose of promoting plaque retention and subsequent local inflammation [22]. The application of the ligatures was performed under 2.5× magnification, using dedicated microsurgical instruments, such as Castroviejo needle holders, microsurgical plyers, dental retraction cords applicators, and periodontal probes (Medesy, Maniago, Italy).

**Figure 1:**
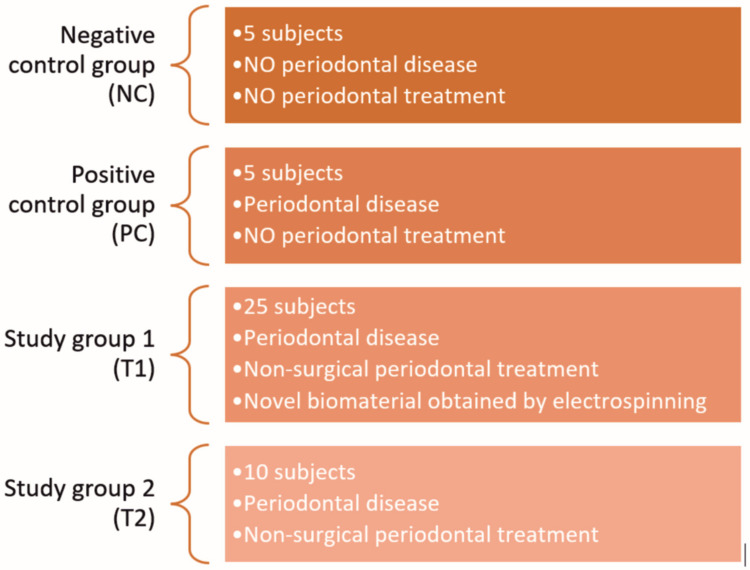
Graphical representation of the specimen’s distribution according to the study design.

The present study included 45 male Wistar rats with similar age, weight, and anthropometrical parameters. The subjects were divided into four groups, as follows (Figure 1): (i) negative control group (NC, 5 subjects): subjects without periodontal disease or treatment; (ii) positive control group (PC, 5 subjects): subjects with induced periodontal disease, but without treatment; (iii) study group 1 (T1, 25 subjects): subjects with induced periodontal disease and combined periodontal treatment, consisting of non-surgical treatment and the local application of the novel electrospun biomaterial; (iv) study group 2 (T2, 10 subjects): subjects with periodontal disease and simple non-surgical periodontal treatment.

**Figure 2:**
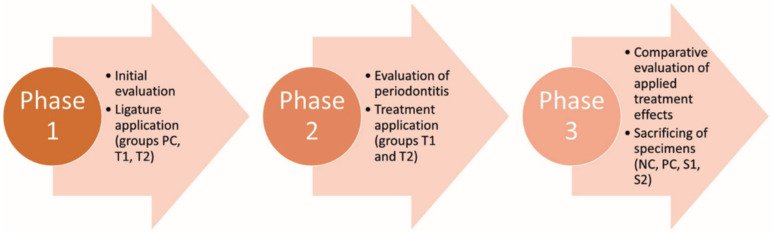
The three different phases of the clinical study.

The experimental protocol was designed in three phases, as follows (Figure 2): Phase 1—initial evaluation of specimens and application of the silk ligature (PC, T1, T2); Phase 2 (14 days after Phase 1)—evaluation of the induced periodontal inflammation, ligature removal and treatment application for animals in groups T1 and T2; Phase 3 (28 days after Phase 2)—clinical evaluation of therapeutic outcomes and animals’ sacrificing to collect tissue specimens for histological examination.

### 2.4. Biological Samples Harvesting and Analysis

Biological samples were collected from all the subjects in all three phases of the experiment. Blood was collected through retro-orbital sinus puncture, using a capillary glass tube. Approximately 1 mL of blood was harvested in sterile vacutainers laced with 3.2% sodium citrate, to prevent coagulation. Immediately after, the vacutainers were centrifuged for 10 min, at 400 rpm, to obtain plasma samples. The processed samples were stored in sterile Eppendorf containers at −80 °C. The ELISA technique was employed to assess plasma levels of IL-1, IL-6 and TNF-α, using commercially available kits: Rat IL-1β ELISA Kit, Elabscience^®^ Biotechnology Inc, Wuhan, Hubei, China, Rat IL-6 ELISA Kit, Elabscience^®^ Biotechnology Inc, Wuhan, Hubei, China, and Rat TNF-α ELISA Kit, Elabscience^®^ Biotechnology Inc, Wuhan, Hubei, China.

Stimulated saliva was harvested under anaesthesia, using a citric acid solution, obtained by dissolving 8 g of citric acid in 20 mL sterile 0.9% sodium chlorite solution. After wiping the excessive citric acid solution from the oral cavity with a sterile gauze, approximately 2 mL of saliva was harvested in sterile Eppendorf containers and stored at −80 °C. The salivary levels of MMP-8 were assessed in all the three phases of the experiment using the commercially available ELISA kit: Rat MMP-8 ELISA Kit, RayBio^®^, Norcross, GA, USA.

### 2.5. Clinical Oral Examination

The periodontal status of the subjects’ lower incisors was clinically evaluated in each phase of the study using the classification proposed by Xu Y. et al. [23]. The parameters used for the evaluation were tooth mobility and the gingival index, with scores between 0 and 3. For tooth mobility, the scores were given as follows: 0 = absence of mobility, 1 = low mobility (in buccal-lingual direction), 2 = moderate mobility (buccal-lingual and mesial-distal directions), and 3 = severe mobility (in axial direction).

The gingival index was clinically assessed by observing the surface texture of the gingiva and the colour changes caused by inflammation, and by gentle probing with a CP-15 periodontal probe (Medesy, Maniago, Italy). The scores were given as follows: 0 = normal aspect of gingiva, 1 = mild inflammation (discreet colour changes, oedema, no bleeding on probing), 2 = moderate inflammation (redness, oedema, change in surface texture, bleeding on probing), and 3 = severe inflammation (tendency of spontaneous bleeding).

### 2.6. Induction of Periodontal Disease

The application of ligatures and the periodontal treatments were performed under general anaesthesia, induced by the intramuscular administration of 0.2 mL 10% Ketamine solution (Vetaketam, Przedsiębiorstwo Wielobranżowe VET-AGRO, Lublin, Poland) and 0.05 mL 2% Xylazine solution (Xylazin Bio, Bioveta, Ivanovice na Hane, Czech Republic). The weight was recorded using an electronic scale, for monitoring the eating habits, the effects of periodontitis, and treatment on the nutritional state of the animals [21].

For treatment standardisation, the PLA nanofiber samples applied in the subjects’ periodontal pockets were individually weighted (Ohaus^®^ ANALYTICAL PLUS, Parsippany, NJ, SUA), and biomaterial samples of 5.79 mg (SD ± 0.1979) were stored in sterile Eppendorf containers.

### 2.7. Treatment Administration

The ligatures were removed after 14 days, and the treatments were applied. The mechanical debridement in groups T1 and T2, consisting in the removal of plaque, calculus, and necrotic cementum from the periodontal pockets, was performed using a 1/2 Gracey curette (Hu-Friedy, Chicago, IL, USA), modified by sharpening with a dedicated device (Sidekick Sharpener, Hu-Friedy, Chicago, IL, USA) in order to reduce the size of the active part. After the mechanical instrumentation, the biomaterial samples were inserted in the periodontal pockets of the subjects in group T1. No sample of the material containing antibiotics was applied to group T2. The treatment was performed under 6×, 10× and 25× magnification, using a medical microscope (Leica M320, Leica Microsystems GmbH, Wetzlar, Germany).

After application, the periodontal pocket was sealed with a mucoadhesive buccal film, which was spread over the electrospun fibers; the adhesion of the film to the gingival mucosa was optimised by prior hydration with sterile saline solution and, after application, it was maintained due to the contact with saliva.

### 2.8. Histological Evaluation

Phase 3 of the study followed four weeks after the treatments were administered. The animals were sacrificed by administration of anaesthetic overdose. The tissue samples, including the lower incisors with the surrounding bone and soft tissues, were harvested using a scalpel and a diamond bur, under constant saline irrigation. The samples were kept in a 10% formalin solution for 4 h, and then decalcified in trichloroacetic acid for approximately 4 weeks. Subsequently, the samples were dehydrated in ethylic alcohol, clarified in 1-butanol, and included in paraffine blocks. Sections of 5 microns were set onto microscopic slides and coloured using Goldner’s trichrome staining method.

### 2.9. Data Collection and Analysis

The photographic documentation of the present study was conducted using dedicated smartphone cameras and the photographic module of the medical Leica microscope (Leica M320, Leica Microsystems GmbH, Wetzlar, Germany). Data analysis was conducted using Microsoft Excel (Microsoft Corporation, Redmond, WA, USA) and GraphPad Prism 5 (GraphPad Software, San Diego, CA, USA). One-way ANOVA was used to compare the values for the gingival index and the tooth mobility in the different phases, whereas the Student’s t-test was used for comparing the salivary concentrations of MMP-8 and plasmatic concentrations of IL-1, IL-6 and TNF-α.

## 3. Results

Four animals were lost between the second and third phases of the experiment: one in the NC group, and three in the T1 group. The necropsy of the deceased subjects revealed no information on the cause of death. Thus, the cause was theorised to be anesthetic complications.

### 3.1. Onset of Periodontal Disease

**Figure 3:**
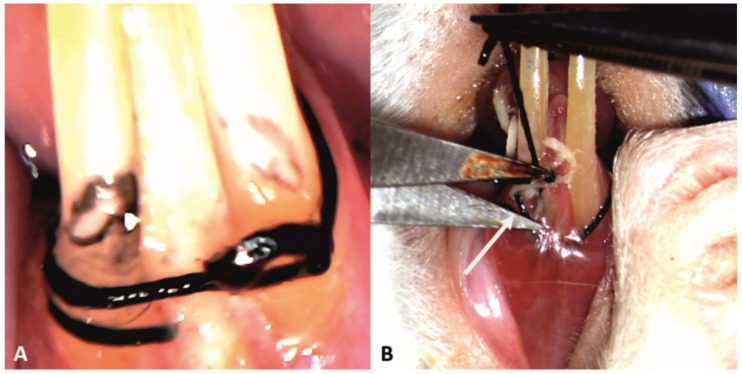
Clinical aspect of the ligatures on the lower incisors of a subject: (**A**) at the moment of application (25× magnification); (**B**) after 14 days, food debris could be found during the ligature removal (white arrow) (10× magnification).

The ligatures were successfully applied to all the subjects in groups PC, T1 and T2 (Figure 3A), and remained in place until removal in the phase 2, 14 days later (Figure 3B).

### 3.2. Treatment

**Figure 4:**
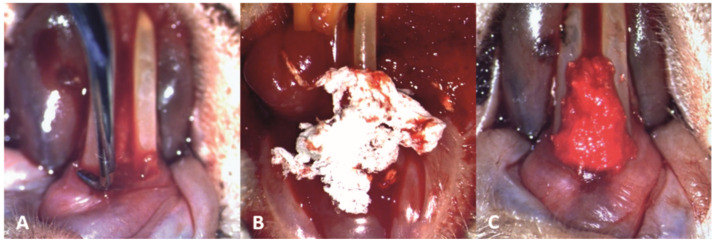
Clinical aspect of the periodontal treatment (10× magnification): (**A**) mechanical debridement of the lower incisors; (**B**) application of the novel biomaterial obtained through electrospinning; (**C**) final aspect of the material applied at a sulcular level and interdentally between the two lower incisors, saturated with blood and promoting local hemostasis.

The mechanical debridement was performed gently to the subjects in groups T1 and T2, using the modified Gracey curette, in order not to cause damage to the inflamed periodontal tissues (Figure 4A). Subsequently, in subjects from the T1 group, the novel biomaterial was applied in the periodontal pockets on the buccal, distal and mesial surfaces of the teeth, and interdentally between the two lower incisors (Figure 4B,C).

**Figure 5:**
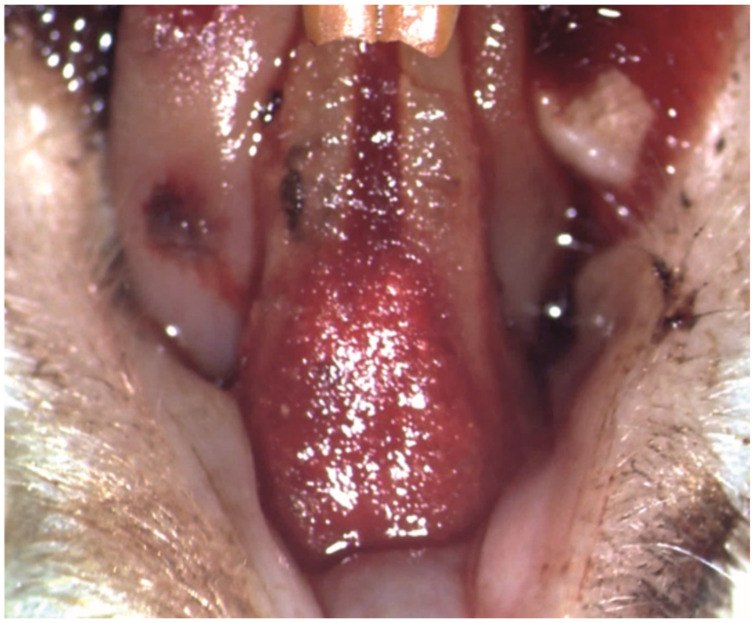
Clinical aspect after the application of the mucoadhesive buccal film covering the periodontal pocket loaded with the electrospun nanofibers.

The periodontal pocket loaded with the new biomaterial was covered with a mucoadhesive buccal film, which became malleable and adhered to the periodontal tissues after hydration with sterile saline solution and the subsequent contact with saliva (Figure 5).

### 3.3. Anthropometric Measurements

After periodontal treatment, the weight gain was higher in groups T1 and T2 compared to PC. However, all the mean values were lower compared to the negative control (Table 1).

### 3.4. Clinical Oral Evaluation

Regarding the tooth mobility, the NC group maintained constant values throughout the study. The groups with induced periodontal disease (PC, T1 and T2) had similar values in the phase 2 of the study, significantly higher compared with phase 1 with PC = 1.6 (SD ± 0.5477), T2 = 1.6 (SD ± 0.5164), and T1 = 1.76 (SD ± 0.4359). In phase 3, the lowest scores in tooth mobility were recorded in group T1 (0.6522, SD ± 0.4870), followed by the PC group (0.75, SD ± 0.5) and T2 group (0.8750, SD ± 0.3536). The one-way ANOVA test of variance for tooth mobility in each periodontitis group show significant differences between the three phases. (Table 2).

The gingival index was null at the beginning of the study in all the four groups and remained constant in the NC group. In phase 2, there were no significant statistical differences between the gingival index scores of all periodontitis groups, with T1 = 1.760 (SD ± 0.4359), PC = 1.6 (SD ± 0.5477) and T2 = 1.6 (SD ± 0.5164). In phase 3, T1 had the lowest gingival index (0.4340, SD ± 0.5069), followed by T2 (0.5, SD ± 0.5345) and PC (0.75, SD ± 0.5345). The one-way ANOVA test of variance conducted for each group with periodontitis revealed significant differences between the three phases (Table 3).

### 3.5. Salivary MMP-8 Levels

**Figure 6:**
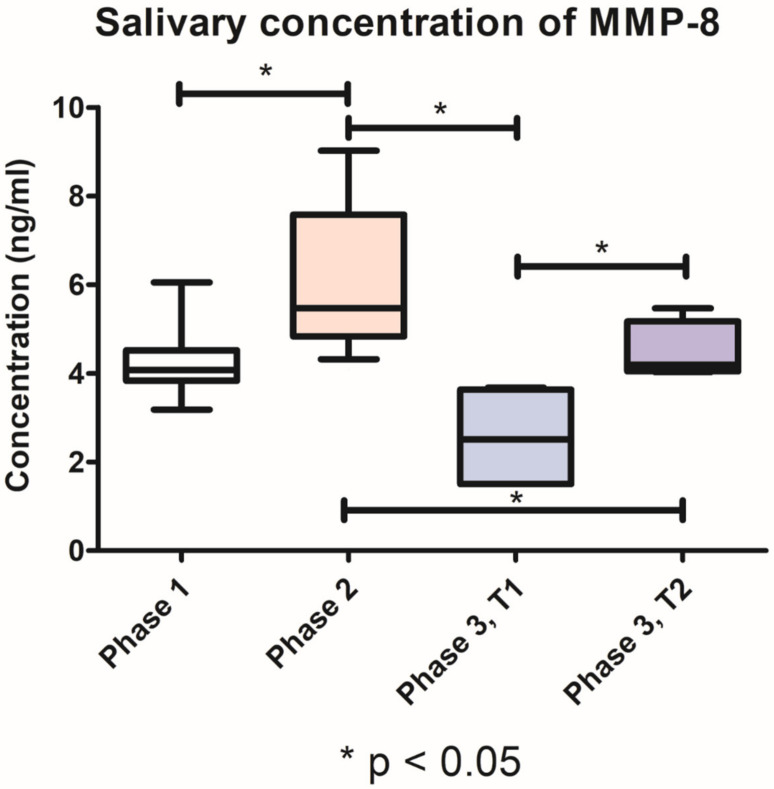
Variation of MMP-8 salivary concentration throughout the three phases of the study.

The collection of biologic fluids presented no difficulties throughout the three phases of the experiment. The salivary concentrations of MMP-8 detected in phases 1 and 2 of the study, respectively, were cumulated, for ease of interpretation and data reporting. In all periodontitis groups, the salivary levels of MMP-8 determined through ELISA technique revealed a significant increase (*p* < 0.05) in phase 2, after the induction of periodontal disease (6.078 ng/mL, SD ± 1.684), compared with phase 1 (4.293 ng/mL, SD ± 0.788). After the periodontal treatment, in phase 3 of the experiment, group T1 had significantly lower levels (*p* < 0.05) of salivary MMP-8 (2.549 ng/mL, SD ± 1.209), compared with group T2 (4.471 ng/mL, SD ± 0.6723). The graphical representation of these values and the relevant statistical differences (t-test) can be found in Figure 6.

### 3.6. Plasma Levels of IL-1, IL-6 and TNF-α

**Figure 7:**
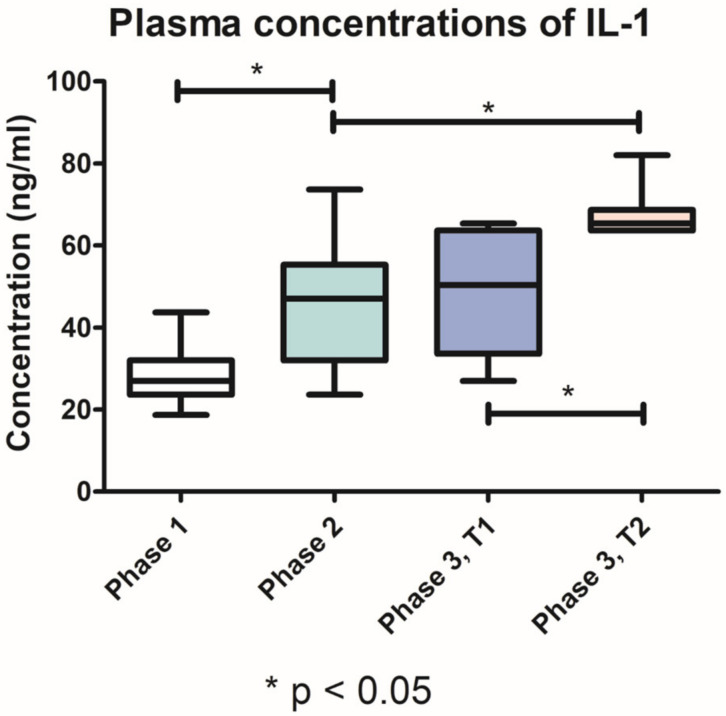
Variation of IL-1 plasma concentrations throughout the three phases of the study.

The plasmatic concentrations of IL-1, IL-6 and TNF-α detected in phases 1 and 2 of the study, respectively, were cumulated, for ease of interpretation and data reporting. The IL-1 plasma levels increased significantly (*p* < 0.05) in phase 2 (44.72 ng/mL, SD ± 13.85) compared with phase 1 (28.54 ng/mL, SD ± 6.470). In the third phase of the study, IL-1 plasma levels in group T1 (49.38 ± 14.72 ng/mL) were significantly lower (*p* < 0.05) than in T2 group (67.95 ng/mL, SD ± 6.445) (Figure 7).

**Figure 8:**
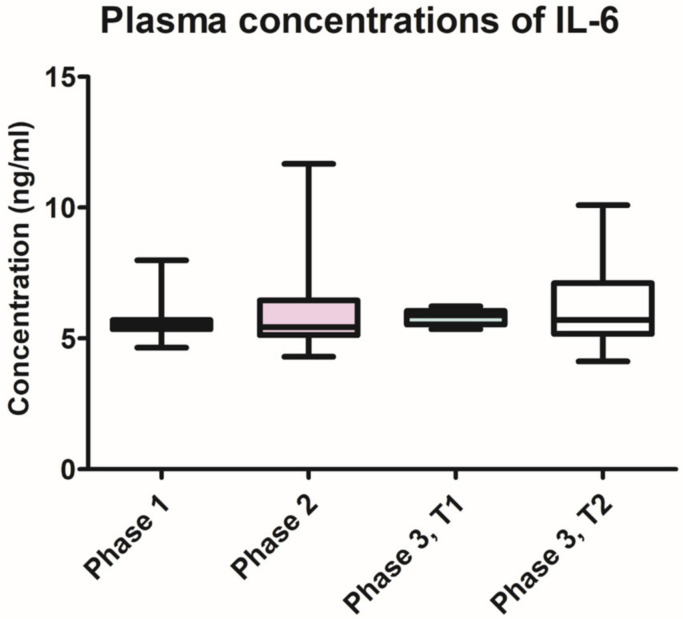
Variation of IL-6 plasma concentrations throughout the three phases of the study.

The IL-6 plasma levels remained constant throughout the different phases of the study, with no statistically significant differences between the three phases, and between the groups (Figure 8). The lowest levels were found in phase 1 of the experiment (5.375 ng/mL, SD ± 0.6669), followed by the ones recorded in phase 3, group T1 (5.827 ng/mL, SD ± 0.3157).

**Figure 9:**
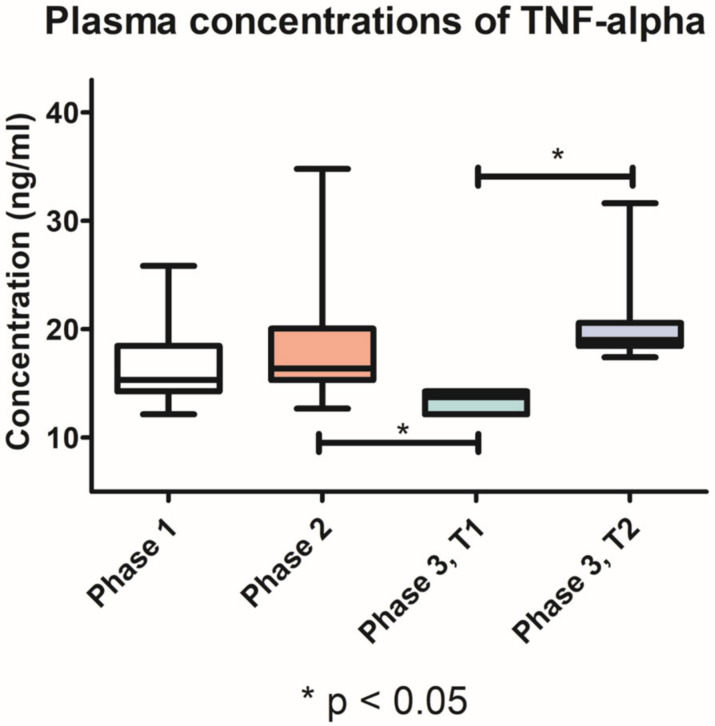
Variation of TNF-α plasma concentrations throughout the three phases of the study.

The TNF-α plasma levels were slightly higher after the induction of periodontitis (18.7 ng/mL, SD ± 5.293) compared with phase 1 (16.67 ng/mL, SD ± 3.509). In T1 group, TNF-α plasma levels were significantly lower (*p* < 0.05) in phase 3 (13.36 ng/mL, SD ± 0.8970), compared with phase 2. Moreover, after treatment, the T1 group had significantly lower (*p* < 0.05) TNF-α plasma levels compared with T2 group (20.88 ng/mL, SD ± 4.851) (Figure 9).

### 3.7. Histological Examination

**Figure 10:**
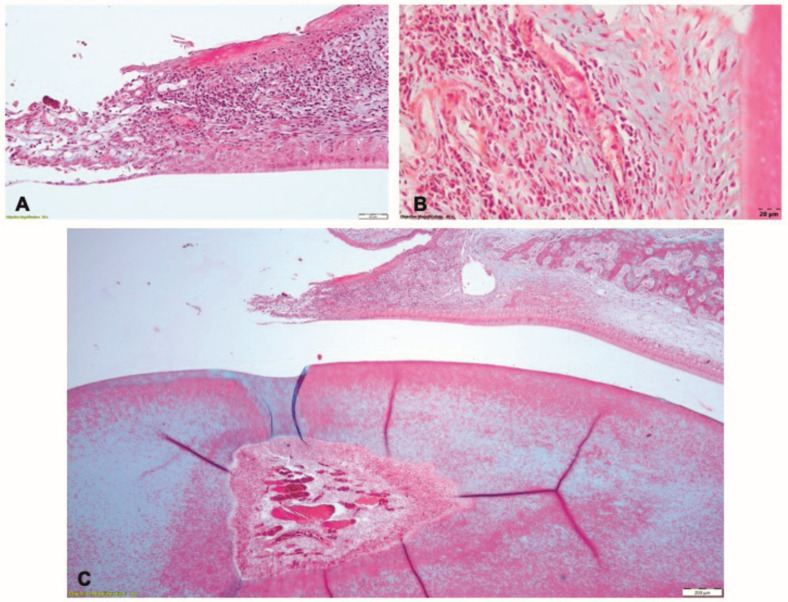
Histological aspect after induction of periodontitis in Phase 2: (**A**) the gingiva with ulcerated epithelium and dense inflammatory infiltrate in the lamina propria; (**B**) rich chronic inflammatory infiltrate in the periodontal ligament; (**C**) resorption of the alveolar bone and detachment of the periodontal ligament; Goldner’s trichrome staining.

After the induction of periodontitis, during phase 2, the gingival epithelium exhibited zones of necrosis and ulceration, associated with a dense chronic inflammatory infiltrate in the underlying lamina propria (Figure 10A). The periodontal ligament contained extensive zones of chronic inflammation, with edema, dilated blood vessels, and inflammatory infiltrate with polymorphonuclears, lymphocytes and macrophages; the fiber groups were detached from the alveolar bone (Figure 10B). The alveolar bone was resorbed, with large areoles and thin trabeculae in the central spongiosa. The blood vessels in the pulp core were congested (Figure 10C).

**Figure 11:**
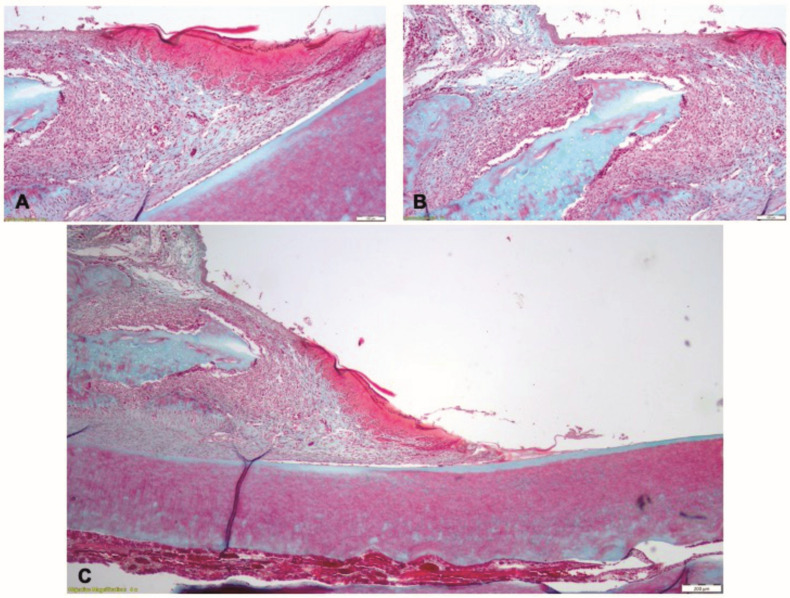
Histological aspect of the T1 group in Phase 3, after treatment with the novel biomaterial: (**A**) the gingival epithelium showing partial healing; (**B**) chronic inflammatory infiltrate in the lamina propria and in the proximity of the alveolar crest; (**C**) persistence of isolated zones of inflammation in the periodontal tissues; Goldner’s trichrome staining.

In Phase 3, in the T1 group, the gingival epithelium exhibited partial healing: in some limited zones, the epithelium was thinner, whereas in other zones the epithelium had normal structure. In the gingival lamina propria, the zones of inflammatory infiltrate were associated with the altered epithelium (Figure 11A). In the coronal part of the periodontal ligament and adjacent to the alveolar crest, the inflammatory infiltrate consisted of polymorphonuclear neutrophils, macrophages with vacuolated cytoplasm, lymphocytes, plasma cells and several multinucleated cells (Figure 11B). The alveolar crest shows resorption on isolated areas. In the profound zone of the periodontal ligament, discrete inflammatory infiltrate with few neutrophil, lymphocytes and plasma cells could be seen (Figure 11C).

**Figure 12:**
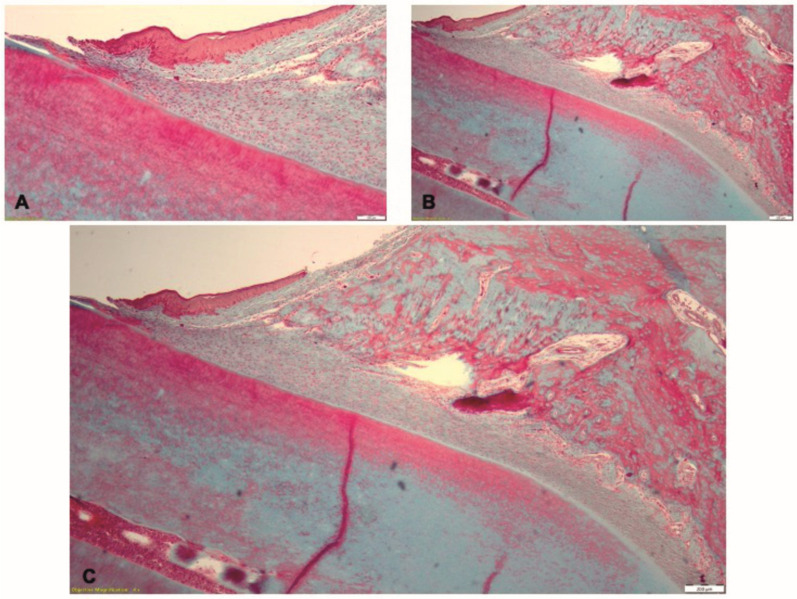
Histological aspect of the T2 group in Phase 3, after treatment by mechanical debridement: (**A**) the gingival epithelium with normal structure; (**B**) isolated zones of inflammatory infiltrate in the gingiva and the periodontal ligament; (**C**) periodontal ligament fibers inserted on the alveolar bone; Goldner’s trichrome staining.

In Phase 3, in T2 group, the histological examination indicates healing of the gingival epithelium (Figure 12A). Few isolated infiltrates with neutrophils and lymphocytes were seen in the lamina propria and the periodontal ligament (Figure 12B). The alveolar bone had normal aspect and the periodontal ligaments inserted on the bone (Figure 12C).

## 4. Discussion

The novel biomaterial obtained through electrospinning was effective for the treatment of experimentally induced periodontal disease, as demonstrated by the improvement of clinical parameters. Moreover, by decreasing the salivary levels of MMP-8 and plasma levels of IL-1 and TNF-α, the biomaterial showed an immunomodulatory effect on the local and systemic biomarkers of the periodontal disease.

The therapeutic effect of the novel biomaterial was tested on male Wistar rats. Female rats were excluded from the study due to the effects of specific hormones on the initiation and progression of periodontal disease [24]. This animal model was chosen, taking into consideration the ease in manipulating the subjects, and the reproductible method for inducing periodontitis [25]. Previous studies recommended the molar area for ligature-induced periodontitis, due to the similarities with the human periodontium both under normal conditions and in periodontal lesions [25,26]. However, an experimental model for the lower incisors was developed by Ionel A et al. [27], with clinical inflammatory aspects present three days after ligature application. Moreover, the histologic examinations conducted after fourteen days revealed the presence of a mixed inflammatory infiltrate and the presence of osteoclasts [27]. For the present study, the lower incisors animal model was considering the accessibility of applying the silk ligatures and the biomaterial used for treatment, as well as for monitoring the therapeutic outcome; additionally, the thin periodontal tissues in this zone are more susceptible to inflammation, thus increasing the reproducibility of the applied therapies.

The main characteristics aimed for the novel biomaterial include resistance during manipulation, the possibility to control the amount of material according to the size of the periodontal pocket, the remanence in the periodontal pocket as well as the slow resorption in contact with oral biofluids (saliva and gingival crevicular fluid). PLA fibers were chosen due to their proven biocompatibility and high mechanical resistance under stress. PLA has a slow degradation rate, occurring through hydrolysis, without toxic residues released during resorption [28]. PLA nanofibers have been previously used for the local application of anti-inflammatory substances in the treatment of periodontitis, as well as for creating 3D-printed scaffolds used for guided bone regeneration [29,30].

Nano-HAP is a bioceramic used for bone regeneration and bone implant surface treatments [31], due to the good biocompatibility, osteoconductive property and the potential to stimulate osteoblastic differentiation; all these mechanisms are essential for alveolar bone healing and potential periodontal regeneration [32]. Moreover, HAP has an affinity for biopolymers, which makes it compatible with PLA [33].

Doxy is a semi-synthetic derivate of tetracycline, known to be efficient against both anaerobic and aerobic pathogens [34]. Furthermore, derivates of tetracycline exhibit immunomodulating effects by inhibiting the activity of collagenases in the gingival chorion, thus enhancing the efficiency of periodontal treatment [34,35,36]. Due to the doxy adsorbed on the surface of the electrospun nanofibers and the slow resorption of PLA, the continuous local release of doxy could be beneficial over time in the treatment of periodontitis.

The in vitro efficacy of the novel doxy-loaded biomaterial obtained through electrospinning has been previously demonstrated against two of the main periodontal pathogens: *A. actinomycetemcomitans* and *P. gingivalis*. The control tests conducted against the two pathogens compared doxy with three other antibiotics frequently used in the adjuvant treatment of periodontitis: amoxicillin, ampicillin and metronidazole. The results indicated that, compared with the other antibiotics, doxy had the largest inhibition zone in *P. gingivalis* culture, and the second largest inhibition zone in *A. actinomycetemcomitans* cultures, after metronidazole [21].

The application of the novel biomaterial obtained through electrospinning caused no technical difficulties, due to the accessibility of the zone where periodontal pathology had been induced and the physical characteristics of the biomaterial. Before application, the material had a cotton-like texture, with a loose fibrillary structure and wide gaps. Upon application in the periodontal sulcus, the material was compacted due to the light force required for the insertion, thus reducing its volume and gaining a denser structure. Moreover, the healing process could be improved due to the blood clot stabilisation by the biomaterial’s imbibition with blood, and by using the newly developed mucoadhesive film to seal the periodontal pocket.

In Wistar rats, the lower incisors exhibit different physiological characteristics when compared with the molars and may normally have a slight mesio-distal mobility. This explains the tooth mobility recorded in nine subjects during the first phase of the study, prior to periodontal lesion induction. The tooth mobility was relatively constant in groups PC, T1 and T2, during the two weeks after ligature application. After treatment, and with the resolution of inflammation, the most significant decrease in tooth mobility was observed in group T1. These results demonstrate the therapeutic potential of the novel electrospun biomaterial by more effectively reducing the periodontal inflammation, compared with the mechanical debridement alone.

Similar findings were observed regarding the gingival index. This parameter evaluates the aspect of the periodontal tissues and, along with the bleeding on probing, is essential for clinical diagnosis in periodontitis [1]. Upon ligature removal, the gingival tissues presented edema, redness and bleeding upon probing. Food debris and localised necrotic gingival epithelium could be seen attached to the ligatures, which further aggravated the inflammation. After the induction of periodontal inflammation, similar gingival index scores were recorded in groups PC, T1 and T2. The most important reduction in gingival index score after treatment application was found in group T1.

The salivary MMP-8 levels significantly increased between phase 1 and 2, suggesting the presence of collagenolytic activity associated with local inflammation. Similar salivary MMP-8 levels were reported by Kasuma N. et al., following ligature-induced periodontitis [37]. After treatment, the lowest salivary MMP-8 levels were observed in group T1 compared with group T2, supporting the positive effect of the tested biomaterial for the resolution of inflammation. The novel biomaterial could be used to limit the periodontal tissues’ destruction and to promote the healing process, thus enhancing the efficacy of the non-surgical periodontal treatment.

The plasma concentrations of IL-1 and TNF-α increased after the induction of periodontitis. These results are consistent with data published by Moradi J. et al. [38]. High concentrations of IL-1 and TNF-α were recorded 10 days after experimentally periodontitis was induced by injecting *Escherichia coli* lipopolysaccharide bilaterally into the palatal gingival tissue [38]. In our study, the plasma concentrations of IL-1 and TNF-α after treatment were significantly lower in group T1 than in group T2. These results demonstrate the local therapeutic effects of the novel biomaterial in periodontal inflammation, as well as the systemic immunomodulatory effect.

Regarding the absence of variation in IL-6 plasma levels throughout the induction and treatment of experimental periodontitis, our results are in contradiction with other data in the literature. Chen D. et al. reported a reduction in serum IL-6 concentrations in rats with induced periodontitis and diabetes mellitus, after periodontal treatment. However, the study design was different, since the rats had been induced by two different pathologies, which could be both responsible for increasing the systemic levels of pro-inflammatory biomarkers [39]. Given these conflicting reports, further research is required to clarify the role of IL-6 in periodontitis.

The histological examination confirmed, in Phase 2, the experimentally-induced periodontal inflammation and revealed the features associated with the different therapeutic approaches in Phase 3. After treatment, the histological aspect of tissue healing in the T1 group was consistent with the clinical parameters. In the periodontal tissues, no nanofiber residue could be seen, suggesting the possibility of complete resorption of the biomaterial. However, a complete resorption of the applied material cannot be concluded, as no chemical identification test was conducted for PLA, nano-HAP or doxy. PLA is known to be biocompatible, but during the resorption process, a chronic inflammatory infiltrate could be seen at the implant site. The persistence of the focal chronic inflammation in the gingival lamina propria and adjacent to the alveolar crest, and the resorption of the alveolar bone were due to the trauma caused by the application of the nanofiber implant. In order to support of the periodontal tissues and to achieve the local concentration of doxy necessary for the therapeutic effect, the size of the matrix samples was predetermined. The physical properties of the biomaterial enabled the condensation of the matrix sample in the periodontal pocket, but due to the excessive size of the matrix and the increased pressure during application, the periodontal tissues were injured. Moreover, the particular physiology of the continuous eruption of the murine incisors and the short period of time between the application of the biomaterial and the histological examination could also explain the incomplete healing of the periodontal tissues. In the T2 group, the non-surgical treatment promoted the periodontal healing through the activation of natural mechanisms; thus, the normal histological aspect of the periodontal tissues could be identified.

The limitations of the present study reside in the difficulties associated with the manipulation of the small periodontal tissues of the subjects, the Wistar rats. The chosen location, however, of the lower incisors facilitated the access for the ligature application and application of the treatment under appropriate magnification.

Another challenge before the evaluation of the novel biomaterial on human subjects refers to the possibility of sterilisation of the samples. For the present study, extreme care was taken in the manufacturing process, using uncontaminated raw materials and taking measures for the sterility of the obtained product, such as using sterile gloves, personal protection equipment and working in a sterile and controlled environment. However, the sterilisation of the final product before the use on human subjects remains a problem, as normal sterilisation processes used in the medical field, such ase heat and ultraviolet light, are thought to be damaging to the final product. Thus, alternate techniques and their influence on the biomaterial, such as gas sterilisation or gamma irradiation, will make the subject of future studies.

## 5. Conclusions

Periodontal disease was successfully induced in rats using the ligature technique on the lower incisors, as confirmed by clinical parameters and the increase in salivary MMP-8 levels and plasma levels of IL-1 and TNF-α, regarded as biomarkers of periodontal disease. The reported decrease in salivary MMP-8 and plasma IL-1 and TNF-α concentrations demonstrate the local and systemic immunomodulatory effect of the novel biomaterial. After periodontal therapy, the levels of the biomarkers varied according to the therapeutic approach. The application of the novel biomaterial obtained through electrospinning in association with the mechanical debridement of the periodontal pockets was more effective in improving the clinical parameters, compared with the non-surgical periodontal treatment alone. Thus, the tested electrospun biomaterial based on PLA and nano-HAP, loaded with doxycycline, could be a strong candidate as an adjuvant to the non-surgical periodontal therapy.

## 6. Patents

Patent application: “Matrix with local antimicrobial and general immunomodulatory effect based on doxycycline encapsulated in polylactic acid and hydroxyapatite nanofibers”, no. OSIM-A/00385/07/05/2022. Aranka Ilea, Reka Barabas, Liliana Antonela Bizo, Oana Cadar, Adina Bianca Boșca, Elena Dinte.

## Figures and Tables

**Table 1:** The mean weights (g) and standard deviations (±SD) of all four groups throughout the three phases of the experiment.

	Phase 1	Phase 2	Phase 3	Mean Increasement between Phases 2 and 3
Negative control (NC)	238 ± 21.71	265.4 ± 30.12	329 ± 22.63	19.33%
Positive control (PC)	266.8 ± 31.53	287.8 ± 34.85	319 ± 25.71	9.78%
Study group T1	240.7 ± 22.33	256.2 ± 26.78	311.3 ± 27.63	17.69%
Study group T2	239.8 ± 23.94	268.6 ± 26.27	303.9 ± 26.65	11.61%

**Table 2:** Tooth mobility scores and standard deviations (± SD) throughout the three phases of the clinical study.

	Phase 1	Phase 2	Phase 3	*p* Value
Negative control (NC)	0.2 ± 0.4472	0.2 ± 0.4472	0.2 ± 0.4472	-
Positive control (PC)	0.2 ± 0.4472	1.6 ± 0.5477	0.75 ± 0.5000	0.0034
Study group T1	0.2 ± 0.4082	1.760 ± 0.4359	0.6522 ± 0.4870	<0.0001
Study group T2	0.2 ± 0.4216	1.6 ± 0.5164	0.8750 ± 0.3536	<0.0001

**Table 3:** Gingival index scores (expressed in mm ± SD—standard deviation) throughout the three phases of the clinical study.

	Phase 1	Phase 2	Phase 3	*p* Value
Negative control (NC)	0	0	0	-
Positive control (PC)	0	1.6 ± 0.5477	0.75 ± 0.5345	0.0003
Study group T1	0	1.760 ± 0.4359	0.4340 ± 0.5069	<0.0001
Study group T2	0	1.6 ± 0.5164	0.5 ± 0.5345	<0.0001
